# Body Composition and Cardiometabolic Risk in Children

**DOI:** 10.1001/jamanetworkopen.2025.35004

**Published:** 2025-10-02

**Authors:** Irene Sequí-Domínguez, Mairena Sánchez-López, Miriam Garrido-Miguel, Monserrat Solera-Martínez, Valentina Díaz-Goñi, Sergio Núñez de Arenas-Arroyo, Pontus Henriksson, Ángel Herraiz-Adillo, Vicente Martínez-Vizcaíno, Eva Rodríguez-Gutiérrez

**Affiliations:** 1 Health and Social Research Center, Universidad de Castilla-La Mancha, Cuenca, Spain; 2 Research Network on Chronicity, Primary Care and Health Promotion (RICAPPS), Cuenca, Spain; 3 Facultad de Educación, Universidad de Castilla-La Mancha, Ciudad Real, Spain; 4 Facultad de Enfermería, Universidad de Castilla-La Mancha, Albacete, Spain; 5 Department of Health, Medicine and Caring Sciences, Linköping University, Linköping, Sweden; 6 Facultad de Ciencias de la Salud, Universidad Autónoma de Chile, Talca, Chile

## Abstract

**Importance:**

Understanding secular patterns in body composition and cardiometabolic risk factors (CMRFs) is essential for identifying early-life determinants of cardiovascular health, guiding public health policies, and developing early prevention strategies.

**Objective:**

To examine secular patterns in the prevalence of underweight, overweight, obesity, and CMRFs among schoolchildren in Spain from 1992 to 2022.

**Design, Setting, and Participants:**

Repeated cross-sectional study analyzing data from 7 time points over a 30-year period in public primary schools from the Cuenca province in Spain. Participants were children aged 8 to 11 years (fourth to fifth grade) from 7 cohorts studied in 1992, 1996, 1998, 2004, 2010, 2018, and 2022. Data were analyzed from April 2024 to May 2025.

**Main outcomes and measures:**

Prevalence of weight status (underweight, normal weight, overweight, and obesity), lipid parameters, glycemic markers, and resting systolic blood pressure (SBP) and diastolic blood pressure (DBP).

**Results:**

In a total of 4280 participants (mean [SD] age, 9.6 [0.7] years; 2137 [50.0%] girls), patterns in the prevalence of weight status show that increases in obesity appear to have slowed down and plateaued since 2018 (13.4%; 95% CI, 11.5% to 15.5% in 2010; 8.1%; 95% CI, 6.0% to 10.5% in 2018; 10.4% 95% CI, 8.1% to 13.2% in 2022; *P* < .001). A statistically significant decrease in mean (SD) total cholesterol (184.6 [27.4] to 160.3 [27.4] mg/dL) and low-density lipoprotein cholesterol (113.6 [24.0] to 90.1 [24.0] mg/dL) was observed, which was reflected in a reduction in non–high-density lipoprotein cholesterol (HDL-C) (125.3 [26.2] to 99.8 [26.1] mg/dL) from 1992 to 2022. Mean (SD) serum HDL-C appear to have peaked from 1998 to 2004 (66.2 [13.3] vs 66.9 [13.3] mg/dL, respectively), and its levels have decreased since then (60.5 [13.4] mg/dL in 2022; *P* < .001). Mean (SD) resting blood pressure showed a substantial decrease over the study period (SBP: 113.5 [9.6] to 101.0 [9.7] mm Hg and DBP: 70.4 [7.2] to 60.7 [7.3] mm Hg; *P* < .001). However, mean (SD) insulin levels show a significant rise from 2004 to 2022 (6.3 [5.3] to 8.7 [5.2] μIU/mL; *P* < .001).

**Conclusions and Relevance:**

This repeated cross-sectional study provides a picture of the evolution of CMRFs in children over the last 30 years, showing that, in Spain, despite the concerning prevalences of excess weight, lipid parameters and blood pressure have improved over the studied period.

## Key Points

**Question:**

How have weight status and cardiometabolic risk factors changed in children from Cuenca, Spain, over the last 30 years?

**Findings:**

In this repeated cross-sectional study of 4280 participants, the prevalence of obesity increased markedly until 2010 but has since plateaued. Significant reductions were observed in total cholesterol, low-density lipoprotein cholesterol, non-high-density lipoprotein cholesterol, and blood pressure, while insulin concentrations have significantly increased since 2004.

**Meaning:**

In this study excess weight remained concerning, but favorable patterns in lipid profiles and blood pressure suggest improvements in cardiovascular health; however, rising insulin levels may indicate emerging risks.

## Teaser

This cross-sectional study evaluates secular patterns in the prevalence of underweight, overweight, obesity, and cardiometabolic risk factors among schoolchildren in Spain from 1992 to 2022.

## Introduction

Atherosclerotic cardiovascular disease (ACVD) remains a leading cause of death and disability worldwide,^1,2^ despite efforts to reduce its impact through risk factor management, improved treatment, and secondary prevention.^3,4^ A robust body of evidence mostly generated over the past few decades supports that cardiometabolic risk factors (CMRFs), such as an altered lipid and glycemic profile, elevated blood pressure, or adiposity parameters, may be preventable.^5,6^ This characteristic is due to CMRFs’ strong association with modifiable health behaviors, particularly a sedentary lifestyle, caloric imbalance, poor nutrition, and unhealthy behaviors.^5,6^

Despite increased awareness, the global burden of ACVD persists, driven by environment and lifestyle changes, such as overconsumption of highly palatable, calorie-dense foods or lack of physical activity (PA), which has spread globally.^7^ Moreover, ACVD appears to be a lifelong companion, since evidence indicates that CMRFs begin in childhood and progress throughout the lifespan.^5,8,9,10,11,12^ However, ACVDs do not manifest clinically until middle and late adulthood, which may lead to a disregard for CMRFs during childhood.^8^

Understanding secular patterns in body composition and CMRFs over extended periods is crucial for identifying early-life determinants of cardiovascular health, guiding public health policies, and developing targeted interventions aimed at preventing the long-term burden of ACVD starting in childhood. Secular patterns of overweight, obesity, and underweight rates in children have been well documented worldwide. Although the studies apply heterogeneous methodologies and span different time periods, they consistently agree on the worrying increase in the prevalence of obesity and overweight from the 1970s, which peaked in the 2000s and seems to be currently stalling.^13,14,15,16,17,18,19,20,21,22,23,24^ However, the associations of cardiometabolic risk with this pattern have received considerably less research attention.

Studies analyzing secular patterns of CMRFs beyond weight status categories in children and adolescents are scarcer, which becomes evident when analyzing different time periods. From the 1980s to the early 2000s a worsening pattern of serum lipid profile, associated or not with an increase in blood pressure, was reported in children and adolescents from Greece and Turkey,^25,26^ in contrast to the improvement in lipid profile and mean blood pressure reported in US adolescents in the same period.^27^ Most studies analyze the 2000s to 2010s period; studies performed in Canada, the United Kingdom, and the US reported a paradoxical evolution of CMRFs, with an improvement in lipid profile and blood pressure despite an increase in adiposity and, in some cases, impaired glycemic parameters.^28,29,30,31^ Fewer studies have evaluated the 2010s to 2020s period. In the US, similar patterns to those observed between 2000 and 2010 were reported.^32^

In Spain, prior research has examined secular patterns in body mass index (BMI) in children with heterogeneous methods and results.^23,33,34,35,36,37,38^ However, no previous study has summarized the prevalence and patterns of weight status categories together with other CMRFs on a serial cross-sectional study including data from the past 30 years. Therefore, the aim of the present study was to examine changes over time in CMRFs, including lipid profile, glycemic parameters, weight status, and blood pressure parameters.

## Methods

### Study Design and Population

This study examined cross-sectional data gathered from studies conducted in 1992, 1996, 1998, 2004, 2010, 2018 and 2022 following the Strengthening the Reporting of Observational Studies in Epidemiology (STROBE) reporting guideline. Of these studies, 3 were cross-sectional studies: the Cuenca Study I (1992; 305 participants), Cuenca Study II (1996; 307 participants), and Cuenca Study III (1998; 276 participants).^39,40^ Data from 2004 (1119 participants),^39^ 2010 (1157 participants),^41^ and 2018 (559 participants)^42^ came from baseline measurements of randomized trials while data from 2022 came from a feasibility trial (557 participants)^43,44^ conducted in the same geographical area, all using similar measurement procedures.

The sample characteristics have been described extensively elsewhere.^39,40,41,42,43,44^ In brief, all the studies were conducted in the same province and the sampling of participants always took place among the same 20 public schools, except for the Cuenca Study, which only included 3 schools from the city of Cuenca. The province of Cuenca is in the center of Spain, and the predominant socioeconomic level is low to middle.

All the studies were designed and conducted in accordance with the Declaration of Helsinki and were approved both by the clinical research ethics committee of the Virgen de la Luz Hospital in Cuenca and by the director and the board of governors of each school.^45^ After obtaining the approval of the director of each school and after informing parents, written approval was requested for their children’s participation. Moreover, the schoolchildren were asked to collaborate with informative talks held class by class.

The sampling methodology was consistent throughout the studies. Randomization occurred at the school level, after which all children in the fourth and fifth grades were invited to participate. To be included in the different projects, children had to meet the following inclusion criteria: literacy in Spanish (or Spanish sign language), absence of serious learning difficulties or physical and mental disorders that could prevent participation in the projects, written consent from a parental or legal guardian, and child verbal consent. Since attendance at primary school is compulsory in our context and most municipalities had only 1 public school, including the entire children’s population in that age group, the representativeness threat was controlled.

#### Anthropometric Measurements

The protocols for data collection were identical for al included studies and self-reported information was obtained on age (years) and sex (female or male). Anthropometric variables were assessed in schools by trained professionals. Weight was measured with the participant lightly dressed without shoes via digital scale with an accuracy of 100 g, and the mean of 2 measurements was taken. Height was assessed using a wall-mounted height rod, with children in stockinged feet, standing straight against the wall so that their spine was vertically aligned with the center of the height rod. The head was positioned in the Frankfurt horizontal plane, and height was measured to the nearest millimeter; final height measure was obtained as the mean of 2 determinations.

We calculated BMI as weight in kilograms divided by the square of the height in meters. Then participants were classified in 4 different groups: underweight, normal weight, overweight, and obesity according to sex- and age-specific criteria of the International Obesity Task Force (IOTF).^46^

Patterns in the prevalence of underweight, normal weight, overweight, and obesity according to the IOTF criteria have been examined in our sample up to 2018 elsewhere.^38^ To maintain continuity with that study, a stacked bar chart is annexed in eFigure 1 in Supplement 1.

#### Lipid and Glycemic Profile

Fasting venous blood was collected at school from the cubital vein between 8:15 and 9:00 am and after a 12-hour fasting period, which was verified through an interview. Serum lipid profiles including total cholesterol (TC), high density lipoprotein cholesterol (HDL-C), low density lipoprotein cholesterol (LDL-C), triglycerides (TG), fasting plasma glucose (FPG), and insulin were determined and analyzed in a Cobas c701 system (Roche Diagnostics).

To calculate remnant cholesterol (REM-C) the following formula was used: REM-C = TC – (LDL-C calculated by the Martin/Hopkins method^47^ + HDL-C). Non-HDL-C was calculated as non-HDL-C = TC – HDL-C.

#### Blood Pressure

Resting systolic blood pressure (SBP) and diastolic blood pressure (DBP) were determined by the mean of 2 measurements taken over a 5-minute period, after a minimum 5-minute rest before the first determination. The children were seated in a quiet and calm environment with their right arm in a semiflexed position at the level of the heart. Blood pressure was obtained with an automatic sphygmomanometer, using 3 sizes of cuffs in accordance with the arm circumference. The final measurement was calculated as the mean of both measurements rounded to the nearest decimal.

### Statistical Analysis

Statistical (Kolmogorov-Smirnov) and graphical (normal probability plots) methods were used to assess the fit to normal distribution of continuous variables. To use parametric methods in all statistical analyses, those variables that did not fit a normal distribution were winsorized using the 1st and the 99th percentiles of their distributions to consider if a fit to normal distribution was possible. Descriptive characteristics including, age, sex, weight, height, and BMI were summarized by survey year. For each weight status category (underweight, normal weight, overweight, and obesity), prevalence and 95% CIs were calculated using the Clopper-Pearson binomial test.

Analysis of covariance (ANCOVA) models were used to provide detailed time-based analysis beyond overall pattern testing by differences over time in raw mean differences in lipid parameters (including TC, HDL-C, LDL-C, TG, non-HDL-C, and REM-C), FPG, insulin, and blood pressure (including SBP and DBP) as dependent variables by survey year (model 0), controlling for age and sex (model 1), and adding BMI as a covariate (model 2). Post hoc pairwise hypotheses were tested for each parameter across the 7 survey years using Bonferroni correction for multiple comparisons to maintain the α error rate at .05.

Furthermore, locally weighted scatterplot smoothed (LOESS) regression was used to display secular patterns in irregularly spaced time points of the variables of interest.^48,49^ Although LOESS is considered a robust method against extreme observations, continuous outcomes were winsorized using the 1st and the 99th percentiles of their distributions to enhance the clarity of the graph.^50^

All statistical analyses were performed using IBM SPSS Statistics version 28.0 (IBM Corp), and a 2-sided *P* < .05 was considered to indicate significance. Data were analyzed from April 2024 to May 2025.

## Results

The general characteristics of a total of 4280 participants (mean [SD] age, 9.6 [0.7] years; 2137 [50.0%] girls) by year of survey are summarized in Table 1. The overall response rate was 77.3% (88.7%, 83.6%, 84.5%, 79.4%, 67.0%, 66.9% and 71.2% in the 1992, 1996, 1998, 2004, 2010, 2018 and 2022 studies, respectively).

**Table 1.** Characteristics of the Study Sample: Children Aged 8 to 11 Years From 1992 to 2022

Characteristic	Participants, mean (SD)						
	1992 (n = 305)	1996 (n = 307)	1998 (n = 276)	2004 (n = 1119)	2010 (n = 1157)	2018 (n = 559)	2022 (n = 557)
Sex, No. (%)							
Female	151 (49.5)	155 (50.5)	138 (50.0)	563 (50.3)	571 (49.3)	291 (52.0)	268 (48.1)
Male	154 (50.5)	152 (49.5)	138 (50.0)	556 (49.7)	586 (50.7)	268 (48.0)	289 (51.9)
Age, y	9.48 (0.54)	9.96 (0.72)	9.39 (0.62)	9.45 (0.66)	9.49 (0.71)	9.56 (0.72)	9.88 (0.59)
Female	9.50 (0.55)	9.88 (0.72)	9.41 (0.61)	9.41 (0.63)	9.48 (0.69)	9.60 (0.69)	9.88 (0.60)
Male	9.46 (0.53)	10.04 (0.72)	9.37 (0.63)	9.4 8 (0.68)	9.51 (0.73)	9.52 (0.74)	9.89 (0.59)
Weight, kg	34.21 (7.17)	38.38 (8.06)	35.81 (7.47)	36.60 (9.34)	37.35 (9.23)	36.65 (10.03)	36.87 (9.63)
Girls	34.18 (7.14)	38.20 (8.86)	35.79 (7.78)	36.37 (9.47)	37.02 (8.80)	36.45 (9.87)	36.85 (9.71)
Boys	34.25 (7.22)	38.57 (7.17)	35.83 (7.19)	36.84 (9.21)	37.68 (9.62)	36.86 (10.21)	36.89 (9.57)
Height, cm	137.72 (6.53)	142.26 (7.56)	139.49 (7.09)	139.69 (6.98)	139.57 (7.00)	140.91 (7.28)	139.93 (7.45)
Girls	137.49 (6.65)	142.06 (7.91)	139.58 (7.75)	139.16 (7.00)	139.62 (7.10)	140.78 (7.46)	139.72 (7.70)
Boys	137.95 (6.42)	142.46 (7.22)	139.39 (6.38)	140.23 (6.92)	139.53 (6.90)	141.06 (7.08)	140.11 (7.21)
BMI^a^	17.91 (2.72)	18.83 (2.83)	18.26 (2.73)	18.57 (3.59)	19.00 (3.69)	18.26 (3.88)	18.63 (3.69)
Girls	17.97 (2.74)	18.76 (3.15)	18.22 (2.81)	18.60 (3.66)	18.85 (3.55)	18.19 (3.71)	18.67 (3.69)
Boys	17.86 (2.71)	18.89 (2.48)	18.30 (2.65)	18.55 (3.53)	19.16 (3.82)	18.35 (4.10)	18.60 (3.68)

Abbreviation: BMI, body mass index.

^a^ BMI calculated as weight in kilograms divided by height in meters squared.

### Patterns of Weight Status From 1992 to 2022

**Figure 1.**
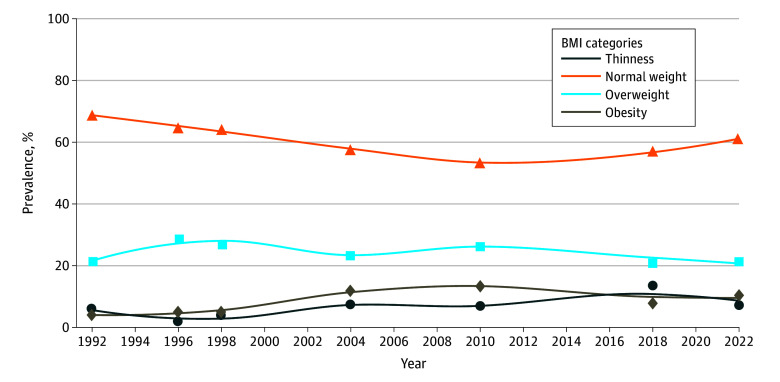
Secular Patterns in the Prevalence of Underweight, Normal Weight, Overweight, and Obesity BMI indicates body mass index, calculated as weight in kilograms divided by height in meters squared.

**Figure 2.**
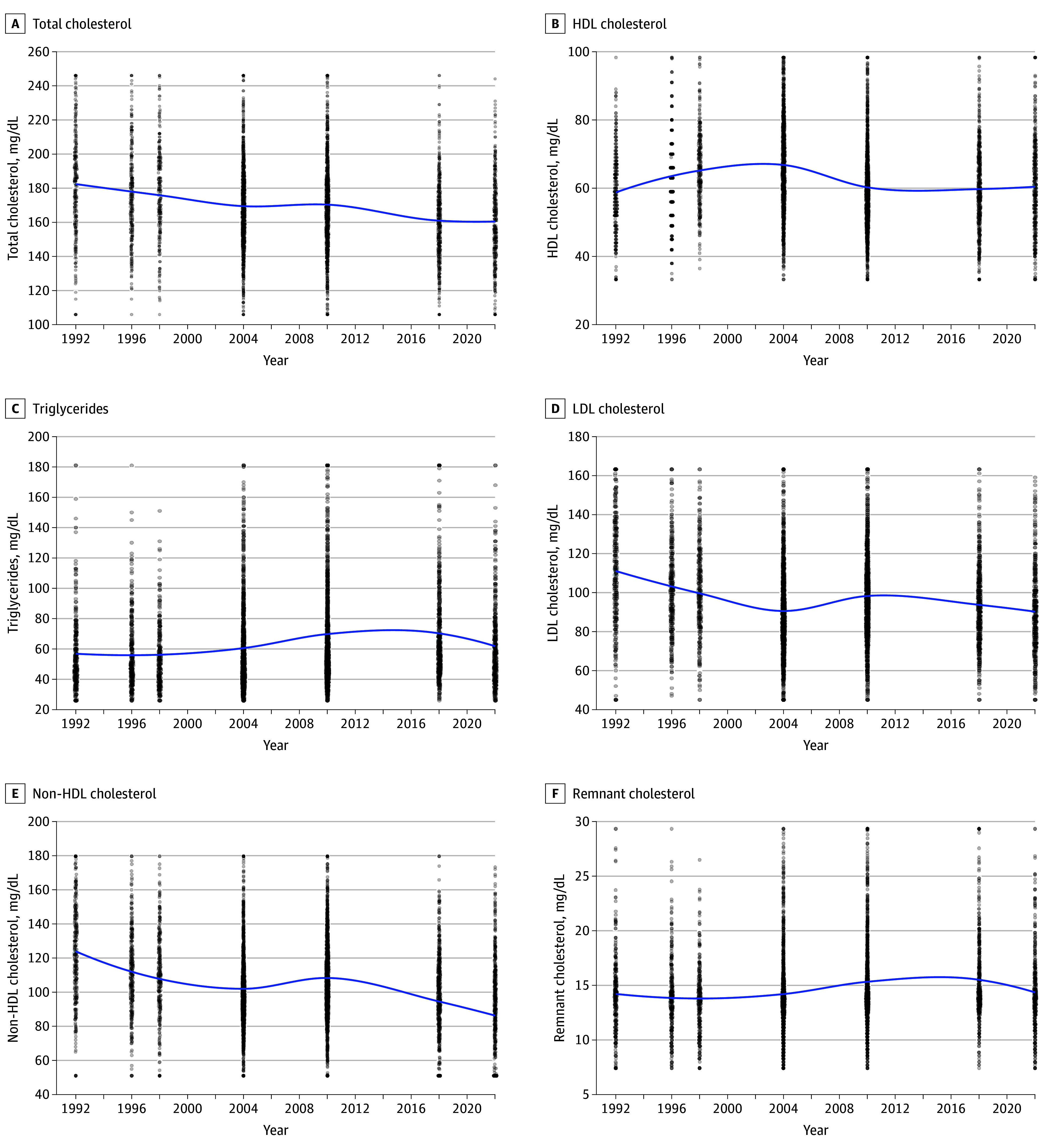
Locally Weighted Regression Pattern of Lipid Parameters HDL indicates high-density lipoprotein; LDL, low-density lipoprotein. To convert high-density lipoprotein cholesterol to millimoles per liter, multiply by 0.259; low-density lipoprotein cholesterol to millimoles per liter, multiply by 0.259; total cholesterol to millimoles per liter, multiply by 0.259; triglycerides to millimoles per liter, multiply by 0.113.

The increasing pattern in the prevalence of overweight and obesity between 1992 and 2010 leveled off and appears to have plateaued in recent years (13.4%; 95% CI, 11.5 to 15.5 in 2010; 8.1%; 95% CI, 6.0 to 10.5 in 2018; 10.4%; 95% CI, 8.1 to 13.2 in 2022 for obesity and 26.2%; 95% CI, 23.7 to 28.8 in 2010; 21.1%; 95% CI, 17.9 to 24.6 in 2018; 21.4%; 95% CI, 18.1 to 24.9 in 2022). Similarly, the prevalence of underweight, which increased after 2004, has recently declined, while the percentage of individuals with a normal weight has begun to rise, reducing the prevalence of underweight, overweight, and obesity (Figure 1; eTable 1 in Supplement 1). Differences by sex are depicted in eFigure 1, eFigure 2, and eTable 2 in Supplement 1 and Figure 2, showing similar patterns.

### Patterns of Lipid Parameters From 1992 to 2022

Figure 2 shows a pattern in lipid parameters including TC, HDL-C, LDL-C, TG, non-HDL-C, and REM-C from 1992 to 2022. Table 2 displays pairwise comparisons between survey years, showing time-based patterns in CMRFs as lipid parameters including TC, HDL-C, LDL-C, TG, non-HDL-C, and REM-C over the 30-year study period. TC demonstrated a consistent declining pattern with significant differences between early years (1992-1998) and all subsequent time points, decreasing from a mean (SD) of 184.6 (27.4) mg/dL in 1992 to 160.3 (27.4) mg/dL in 2022 (to convert TC, LDL-C, HDL-C, and REM-C to millimoles per liter, multiply by 0.0259). Mean (SD) LDL-C showed the most pronounced improvement, with 1992 levels (113.6 [24.0] mg/dL) consistently decreasing to 90.1 (24.0) mg/dL (*P* < .001), which was reflected in a reduction pattern in non–high-density lipoprotein cholesterol (HDL-C) (125.3 [26.2] to 99.8 [26.1] mg/dL) from 1992 to 2022. Mean (SD) serum HDL-C appeared to have peaked in 1998 to 2004 (66.2 [13.3] and 66.9 [13.3] mg/dL, respectively), before declining to levels similar to the 1990s (59.5 [13.3] mg/dL in 1992 to 60.5 [13.4] mg/dL in 2022).

**Table 2.** Levels of Lipid, Glycemic, and Blood Pressure Parameters From 1992 to 2022^a^

Measurement	1992 (n = 305)	1996 (n = 307)	1998 (n = 276)	2004 (n = 1119)	2010 (n = 1157)	2018 (n = 559)	2022 (n = 557)
Lipid profile							
TC, mg/dL	184.8 (27.4)	176.1 (27.7)	178.4 (27.4)	169.4 (27.4)	169.0 (27.4)	161.0 (27.4)	160.5 (27.7)
HDL-C, mg/dL	58.6 (12.6)	62.8 (12.7)	65.7 (12.5)	66.9 (12.6)	60.2 (12.5)	59.4 (12.6)	60.4 (12.6)
LDL-C, mg/dL	114.3 (23.9)	102.1 (24.2)	101.4 (23.8)	90.4 (23.9)	96.8 (23.9)	93.9 (23.8)	90.4 (24.1)
TG, mg/dL	60.3 (28.2)	55.9 (28.0)	56.4 (28.2)	61.0 (28.2)	67.6 (28.3)	71.7 (28.2)	61.6 (28.4)
Non-HDL-C, mg/dL	126.2 (25.1)	113.3 (24.5)	112.7 (24.2)	102.5 (24.1)	108.8 (24.3)	101.6 (24.0)	100.1 (24.3)
REM-C, mg/dL	14.6 (3.8)	13.8 (3.8)	13.8 (3.8)	14.3 (4.0)	15.3 (3.7)	15.7 (3.7)	14.3 (3.8)
Glycemic parameters							
FPG, mg/dL	NA	NA	NA	86.3 (7.0)	83.6 (6.7)	87.6 (6.8)	84.3 (7.0)
Insulin, μIU/mL	NA	NA	NA	6.4 (4.6)	7.8 (4.4)	8.4 (4.4)	8.7 (4.6)
Blood pressure							
SBP, mm Hg	114.1 (9.3)	118.5 (9.3)	110.0 (9.3)	107.2 (9.4)	101.0 (9.2)	99.0 (9.2)	100.4 (9.4)
DBP, mm Hg	70.8 (7.0)	63.9 (7.0)	69.0 (7.0)	65.1 (7.0)	62.2 (6.8)	63.8 (6.9)	60.6 (7.1)

Abbreviations, DBP, diastolic blood pressure; FPG, fasting plasma glucose; HDL-C, high density lipoprotein cholesterol; LDL-C, low density lipoprotein cholesterol; NA, not applicable; REM-C, remnant cholesterol; SBP, systolic blood pressure; TC, total cholesterol; TG, triglycerides.

SI conversions: To convert FPG to millimoles per liter, multiply by 0.0555; HDL-C to millimoles per liter, multiply by 0.259; insulin to picomoles per liter, multiply by 6.945; LDL-C to millimoles per liter, multiply by 0.259; TC to millimoles per liter, multiply by 0.259; TG to millimoles per liter, multiply by 0.113.

^a^ Values adjusted by age, sex and body mass index. All analysis of covariance models displayed were statistically significant (*P* < .001), superscript letters indicate statistical significance (*P* < .05) between survey year for post hoc tests using the Bonferroni comparisons. eTable 6 in Supplement 1 shows which values were significantly different when compared with other years.

Despite the decreasing pattern in other lipid parameters, and similarly to HDL-C levels, mean (SD) TGs and REM-C seem to have peaked between 2010 and 2018 (TGs: 68.7 [31.0] mg/dL in 2010 and 71.0 [30.9] mg/dL in 2018 [to convert TGs to millimoles per liter, multiply by 0.0113]; REM-C: 15.4 [4.0] mg/dL in 2010 and 15.6 [4.2] in 2018), and by 2022, they appear to be decreasing back to previous levels (TGs: 57.5 [31.0] mg/dL in 1992 and 62.0 [30.8] mg/dL in 2022; REM-C: 14.2 [4.1] mg/dL in 1992 and 14.4 [4.2] in 2022). These results were maintained even when the ANCOVA models adjusted for age, sex, and BMI as displayed in Table 2 (complete ANCOVA models are reported in eTable 3 in Supplement 1). Patterns in serum lipid parameters stratified by IOTF categories are displayed in eFigure 3 in Supplement 1 showing consistent patterns.

### Patterns of Glycemic Parameters From 2004 to 2022

**Figure 3.**
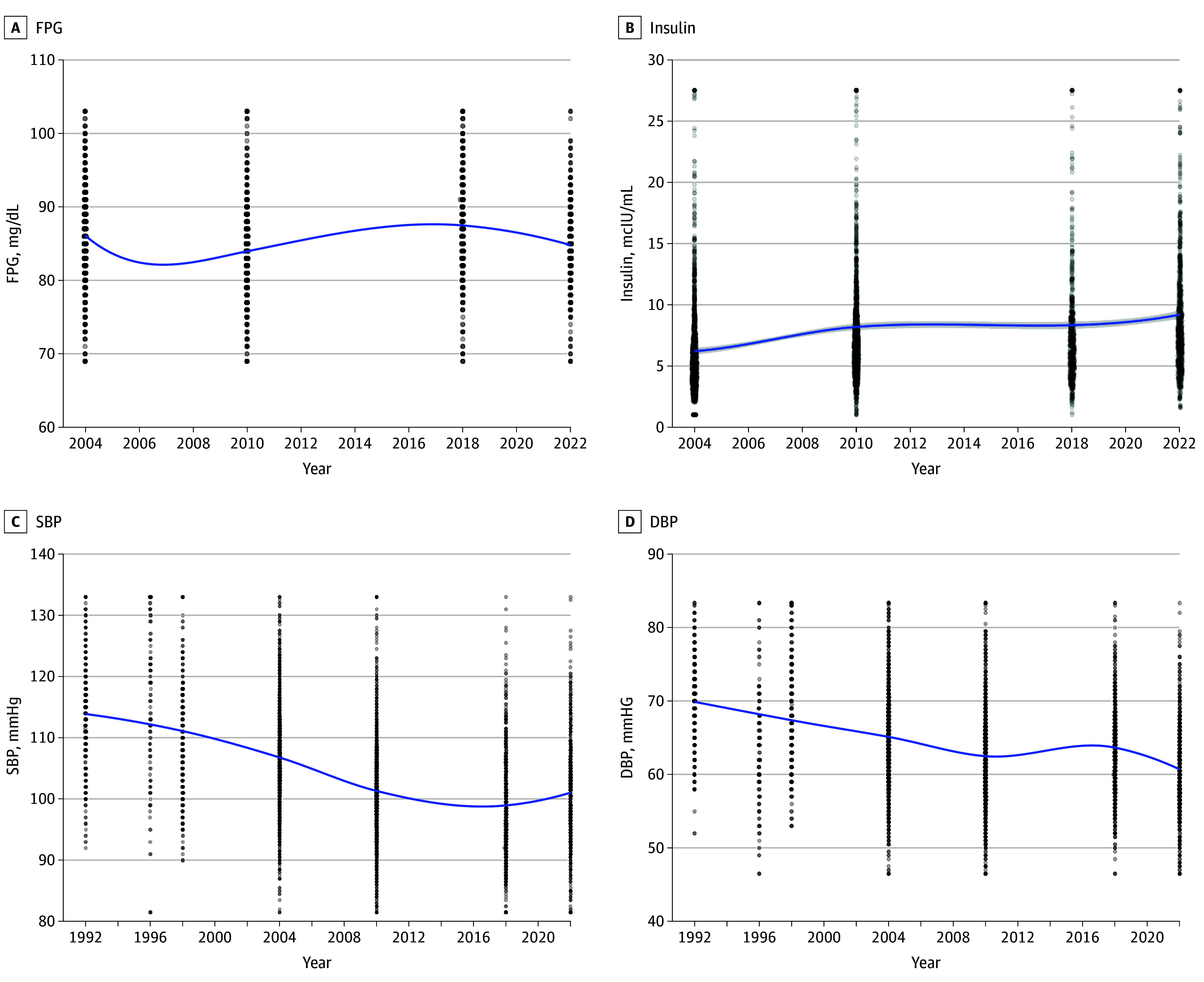
Locally Weighted Regression Pattern of Glycemic and Blood Pressure Parameters Through the Study Period DBP indicates diastolic blood pressure; FPG, fasting plasma glucose; SBP, systolic blood pressure. SI conversions: To convert FPG to nanomoles per liter, multiply by 0.0555; insulin to picomoles per liter, multiply by 6.945.

Data on glycemic parameters, including FPG and insulin, started to be collected in the 2004 survey (Figure 3). Patterns in mean (SD) FPG showed modest but significant variations peaking in 2018 (2004: 86.2 [7.0] mg/dL; 2010: 83.6 [7.1] mg/dL; 2018: 87.6 [7.1] mg/dL; 2022: 84.8 [7.0] mg/dL; *P* < .001 [to convert to millimoles per liter, multiply by 0.0555]) while insulin levels increased significantly (2004: 6.3 [5.3]μIU/mL to 2022: 8.7 [5.2] μIU/mL; *P* < .001 [to convert to picomoles per liter, multiply by 6.945]).

Statistically significant differences were found for both parameters through the years, even when the ANCOVA models were adjusted for age, sex, and adding BMI as displayed in Table 2 (complete ANCOVA models are reported in eTable 4 in Supplement 1). Patterns in glycemic parameters stratified by IOTF categories are displayed in eFigure 4 in Supplement 1 showing consistent patterns.

### Patterns of Blood Pressure Parameters From 1992 to 2022

Mean (SD) resting SBP and DBP levels (Figure 3) showed a substantial decrease over the study period (SBP: 113.5 [9.6] in 1992 to 101.0 [9.7] mm Hg in 2022 and DBP: 70.4 [7.2] in 1992 to 60.7 [7.3] mm Hg in 2022; *P* < .001). The decrease remained when adjusting by age, sex, and adding BMI (*P* < .001), as shown in Table 2 (complete ANCOVA models are reported in eTable 5 in Supplement 1). Patterns in blood pressure parameters stratified by IOTF categories are displayed in eFigure 5 in Supplement 1 showing consistent patterns.

## Discussion

Our results provide an updated picture of the prevalence of weight status and secular patterns in CMRFs in schoolchildren from Cuenca, Spain, from 1992 to 2022. Our data show that the percentage of children in the normal weight category is now increasing for the first time since the 2000s. This increase comes at a time when the prevalence of overweight and obesity appears to be plateauing, although it remains at a concerning rate of around 30%; similarly, the prevalence of underweight is declining after peaking in 2018. As for CMRFs, while the prevalence of excess weight remains concerning, favorable patterns in lipid profiles and blood pressure were observed, while insulin showed a consistently increasing pattern.

According to data from the World Health Organization, in Europe, excess weight is still increasing in 2022, whereas underweight remains stable.^51,52^ Yet, focusing on the Southern European region—which includes Greece, Italy, Portugal, Slovenia, and Spain, historically among those with the highest childhood obesity rates in Europe—excess weight prevalences are shown to be stalling in the population aged between 5 and 19 years .^51,52,53^ Our study, by updating the prevalence rates of weight status in our population,^38^ shows that recent rates of overweight and obesity are similar to those reported in the latest studies of the Spanish population.^54,55^ However, this potential plateauing pattern of the figures of excess weight is not consistent with the pattern reported in the review by Bravo-Saquicela et al.^56^ They showed an increase in the prevalence of excess body weight patterns between 1999 and 2010 and 2011 and 2021, which may have been affected by the time frames selected for their study.

In addition, our findings are consistent with the data from the ALADINO 2023 study in a population based sample of Spanish children, which reported a decrease in overweight and obesity rates from 2019 to 2023, concluding that although values remained high, there was a decrease in overweight and obesity rates in favor of the normal weight category in the last years.^37,57^ The main difference between our study and the ALADINO study lies in the follow-up period, as our research began in 1992, providing a 30-year perspective, whereas the ALADINO study started in 2007. Although the ALADINO study draws from a nationally representative sample of children across Spain, its estimates may be affected by a high nonresponse rate (60%). Nonetheless, when the data from both studies are considered together, they provide complementary evidence that reinforces the notion that the prevalence of overweight and obesity in Spain is stabilizing.^57^

In the city of Cuenca, Spain, CMRFs have been monitored since 1992, and given the stability of the participating schools, these data provide a comprehensive picture of the evolution of CMRFs over the last 30 years. Alongside the increase and subsequent plateau of excess weight, lipid profiles and blood pressure seem to have consistently improved over the years. Notably, TG and REM-C appear to be returning to levels observed before the 2008 financial crisis, a period marked by worsening socioeconomic inequalities that contributed to a decline in lifestyle quality, particularly in healthy eating behaviors and mental health, subsequently leading to a rise in lipid profiles.^58^ These findings are consistent with studies conducted in different countries.^26,27,32,59^ However, the increasing, albeit modest, pattern in plasma insulin levels, which are considered a sensitive indicator of insulin resistance even in children without elevated FPG, is a cause for concern.^60^

The scarcity of longitudinal data on CMRFs in children since 2010 presents a challenge for contextualizing our findings. Nevertheless, several hypotheses can be put forward to explain these findings. First, although stabilizing, the high BMI patterns do not seem to be causing a worsening of the lipid profile and blood pressure patterns, suggesting that other factors such as muscular strength may be influencing this relationship. This paradoxical improvement in certain CMRFs may be attributed to the implementation of global policy initiatives aimed at mitigating the childhood obesity epidemic by promoting healthy nutrition and physical activity, such as the Strategy for Nutrition, Physical Activity and Obesity Prevention,^61^ as the available evidence on this issue has increased significantly over the last 3 decades.^62^

### Limitations and Strengths

It is important to consider several limitations when interpreting the results of this study. First, our analyses were limited to children aged 8 to 11 years from Cuenca, Spain, and thus, the results should be interpreted with caution when extrapolating to other populations. Second, the cohort effect limits the ability to generalize these findings to other birth cohorts. Third, despite the fact that the analytical determinations were always carried out in the same laboratory following the same protocols, due to the long period studied and evolution of measurement methods, measurements might have been compromised. Fourth, given that participants were aged between 8 and 11 years, maturation status may have acted as a confounding variable, particularly among girls, due to the earlier onset of pubertal development in this group. However, maturation stage (eg, Tanner stage) was not included in the analysis, as these data were not collected in earlier project phases. Fifth, there were limitations in comparing our results with those of other studies analyzing CMRFs secular patterns due to differences in population characteristics, measurement criteria, and time periods.

Our findings should be interpreted considering the study design. Although the data are not representative in the traditional sense, the 30-year repeated cross-sectional approach allows us to address the secular patterns. Future studies combining representative sampling with longitudinal follow-up could further clarify age-related trajectories and age, period, and cohort effects.

Notwithstanding these constraints, our repeated cross-sectional design over 3 decades is based on data that remain consistent over time in terms of population and measurement methods. Despite its limited generalizability to the broader population, the consistency of the sampling frame across waves ensures internal validity for pattern analyses. Hence, these data are uniquely suited to examine long-term patterns in cardiometabolic parameters among children in Spain, providing a comprehensive picture of the evolution of CMRFs in children over the last 30 years.

## Conclusions

The present study provides an updated picture of the prevalence of weight status categories and the secular pattern of CMRFs in schoolchildren in Cuenca, Spain, from 1992 to 2022. The results support that in Spain, the concerning pattern in childhood overweight and obesity prevalence has peaked, and, despite remaining noteworthy, favorable patterns in lipid profiles and blood pressure suggest improvements in overall cardiometabolic risk; however, rising insulin levels may indicate emerging risks associated with insulin resistance and future metabolic disorders.

## Notes

Supplement 1. eTable 1. Patterns in Prevalence of Weight Status Categories, (Percentage and 95% CI) From 1992 to 2022 eTable 2. Patterns in Prevalence of Weight Status Categories (Percentage and 95% CI) From 1992 to 2022 Divided by Sex eTable 3. Levels of Lipid Parameters From 1992 to 2022 eTable 4. Levels of Glycemic Parameters From 2004 to 2022 eTable 5. Levels in Blood Pressure Parameters From 1992 to 2022 eTable 6. Levels of Lipid, Glycemic, and Blood Pressure Parameters From 1992 to 2022 eFigure 1. Prevalence and Patterns of Underweight, Normal Weight, Overweight, and Obesity From 1992 to 2022 eFigure 2. Secular Patterns in Prevalence of Underweight, Normal Weight, Overweight, and Obesity From 1992 to 2022 by Sex eFigure 3. Locally Weighted Regression (LOESS) Patterns of Lipid Parameters From 1992 to 2022 by IOTF Categories eFigure 4. Locally Weighted Regression (LOESS) Patterns of Fasting Plasma Glucose (FPG) and Insulin Levels From 2004 to 2022 eFigure 5. Locally Weighted Regression (LOESS) Patterns of Systolic Blood Pressure (SBP) and Diastolic Blood Pressure (DBP) From 1992 to 2022

Supplement 2. Data Sharing Statement
