# Supplementary material for: Body Composition and Cardiometabolic Risk in Children
Source: JAMA Netw Open. 2025 Oct 2;8(10):e2535004. doi: 10.1001/jamanetworkopen.2025.35004 (PMC12492057; doi:10.1001/jamanetworkopen.2025.35004)
Supplement: Supplement 1. — eTable 1. Patterns in Prevalence of Weight Status Categories, (Percentage and 95% CI) From 1992 to 2022 eTable 2. Patterns in Prevalence of Weight Status Categories (Percentage and 95% CI) From 1992 to 2022 Divided by Sex eTable 3. Levels of Lipid Parameters From 1992 to 2022 eTable 4. Levels of Glycemic Parameters From 2004 to 2022 eTable 5. Levels in Blood Pressure Parameters From 1992 to 2022 eTable 6. Levels of Lipid, Glycemic, and Blood Pressure Parameters From 1992 to 2022 eFigure 1. Prevalence and Patterns of Underweight, Normal Weight, Overweight, and Obesity From 1992 to 2022 eFigure 2. Secular Patterns in Prevalence of Underweight, Normal Weight, Overweight, and Obesity From 1992 to 2022 by Sex eFigure 3. Locally Weighted Regression (LOESS) Patterns of Lipid Parameters From 1992 to 2022 by IOTF Categories eFigure 4. Locally Weighted Regression (LOESS) Patterns of Fasting Plasma Glucose (FPG) and Insulin Levels From 2004 to 2022 eFigure 5. Locally Weighted Regression (LOESS) Patterns of Systolic Blood Pressure (SBP) and Diastolic Blood Pressure (DBP) From 1992 to 2022 [file jamanetwopen-e2535004-s001.pdf]

Supplemental Online Content

Sequí-Domínguez I, Sánchez-López M, Garrido-Miguel M, et al. Body composition and cardiometabolic risk in children. *JAMA Netw Open*. 2025;8(10):e2535004. doi:10.1001/jamanetworkopen.2025.35004

eTable 1. Patterns in Prevalence of Weight Status Categories, (Percentage and 95% CI) From 1992 to 2022

eTable 2. Patterns in Prevalence of Weight Status Categories (Percentage and 95% CI) From 1992 to 2022 Divided by Sex

eTable 3. Levels of Lipid Parameters From 1992 to 2022

eTable 4. Levels of Glycemic Parameters From 2004 to 2022

eTable 5. Levels in Blood Pressure Parameters From 1992 to 2022

eTable 6. Levels of Lipid, Glycemic, and Blood Pressure Parameters From 1992 to 2022

eFigure 1. Prevalence and Patterns of Underweight, Normal Weight, Overweight, and Obesity From 1992 to 2022

eFigure 2. Secular Patterns in Prevalence of Underweight, Normal Weight, Overweight, and Obesity From 1992 to 2022 by Sex

eFigure 3. Locally Weighted Regression (LOESS) Patterns of Lipid Parameters From 1992 to 2022 by IOTF Categories

eFigure 4. Locally Weighted Regression (LOESS) Patterns of Fasting Plasma Glucose (FPG) and Insulin Levels From 2004 to 2022

eFigure 5. Locally Weighted Regression (LOESS) Patterns of systolic Blood Pressure (SBP) and Diastolic Blood Pressure (DBP) From 1992 to 2022

This supplemental material has been provided by the authors to give readers additional information about their work.

© 2025 Sequí-Domínguez I et al. *JAMA Network Open*.

eTable 1. Patterns in Prevalence of Weight Status Categories, (Percentage and 95% CI) From 1992 to 2022

	1992	***1996***	***1998***	***2004***	***2010***	***2018***	***2022***
n	305	307	276	1119	1157	559	557
Underweight	5.9	1.6	4.0	7.2	7.0	13.6	7.4
	(3.7, 9.0)	(0.6, 3.5)	(2.1, 6.8)	(5.8, 8.9)	(5.6, 8.6)	(10.9, 16.6)	(5.4, 9.8)
Normal weight	68.9	64.5	64.1	57.6	53.4	57.2	60.9
	(63.5, 73.9)	(59.0, 69.7)	(58.3, 69.6)	(54.7, 60.5)	(50.5, 56.3)	(53.1, 61.3)	(56.8, 64.9)
Overweight	21.3	28.7	26.8	23.3	26.2	21.1	21.4
	(17.0, 26.2)	(23.8, 33.9)	(21.8, 32.3)	(20.9, 25.9)	(23.7. 28.8)	(17.9, 24.6)	(18.1, 24.9)
Obesity	3.9	5.2	5.1	11.8	13.4	8.1	10.4
	(2.2, 6.6)	(3.1, 8.1)	(2.9, 8.1)	(10.0, 13.8)	(11.5, 15.5)	(6.0, 10.5)	(8.1, 13.2)

eTable 2. Patterns in Prevalence of Weight Status Categories (Percentage and 95% CI) From 1992 to 2022 Divided by Sex

	1992	***1996***	***1998***	***2004***	***2010***	***2018***	***2022***
N boys	154	152	138	556	587	269	289
Underweight	6.5	0.7	2.9	7.6	7.3	11.9	5.5
	(3.4, 11.2)	(0.1, 3.0)	(1.0, 6.7)	(5.6, 10.0)	(5.4, 9.6)	(8.4, 16.2)	(3.3, 8.6)
Normal weight	70.8	64.5	63.8	58.1	51.4	57.6	64.4
	(63.3, 77.5)	(56.6, 71.8)	(55.5, 71.4)	(54.0, 62.1)	(47.4, 55.5)	(51.7, 63.4)	(58.7, 69.7)
Overweight	18.2	32.2	29.7	21.6	26.9	21.9	19.4
	(12.7, 24.8)	(25.2, 39.9)	(22.6, 37.7)	(18.3, 25.1)	(23.4, 30.6)	(17.3, 27.2)	(15.1, 24.2)
Obesity	4.5	2.6	3.6	12.8	14.3	8.6	10.7
	(2.1, 8.7)	(0.9, 6.1)	(1.4, 7.8)	(10.2, 15.7)	(11.7, 17.3)	(5.6, 12.3)	(7.6, 14.7)
N girls	151	155	138	563	570	290	268
Underweight	5.3	2.6	5.1	6.9	6.7	15.2	9.3
	(2.5, 9.7)	(0.9, 6.0)	(2.3, 9.7)	(5.0, 9.2)	(4.8, 8.9)	(11.4, 19.6)	(6.3, 13.2)
Normal weight	66.9	64.5	64.5	57.2	55.4	56.9	57.1
	(59.1, 74.0)	(56.8, 71.7)	(56.3, 72.1)	(53.1, 61.2)	(51.3, 59.5)	(51.2, 62.5)	(51.1, 62.9)
Overweight	24.5	25.2	23.9	25.0	25.4	20.3	23.5
	(18.2, 31.8)	(18.8, 32.4)	(17.4, 31.5)	(21.6, 28.7)	(22.0, 29.1)	(16.0, 25.3)	(18.7, 28.9)
Obesity	3.3	7.7	6.5	10.8	12.5	7.6	10.1
	(1.3, 7.1)	(4.3, 12.7)	(3.3, 11.6)	(8.5, 13.6)	(9.9, 15.4)	(5.0, 11.1)	(6.9, 14.1)

eTable 3. Levels of Lipid Parameters From 1992 to 2022

	1992	***1996***	***1998***	***2004***	***2010***	***2018***	***2022***

© 2025 Sequí-Domínguez I et al. *JAMA Network Open*.

		(A)	(B)	(C)	(D)	(E)	(F)	(G)
	n	305	307	276	1119	1157	559	557
TC	M0	184.6 ± 27.4<br>B,D,E,F,G	175.9 ± 27.4<br>A,D,E,F,G	178.4 ± 27.4<br>D,E,F,G	169.5 ± 27.4<br>A,B,C,F,G	169.2 ± 27.4<br>A,B,C,F,G	160.9 ± 27.4<br>A,B,C,D,E	160.3 ± 27.4<br>A,B,C,D,E
	M1	184.6 ± 27.4<br>B,D,E,F,G	176.1 ± 27.7<br>A,D,E,F,G	178.3 ± 27.4<br>D,E,F,G	169.4 ± 27.5<br>A,B,C,F,G	169.2 ± 27.4<br>A,B,C,F,G	160.9 ± 27.4<br>A,B,C,D,E	160.5 ± 27.7<br>A,B,C,D,E
	M2	184.8 ± 27.4<br>B,D,E,F,G	176.1 ± 27.7<br>A,D,E,F,G	178.4 ± 27.4<br>D,E,F,G	169.4 ± 27.4<br>A,B,C,F,G	169.0 ± 27.4<br>A,B,C,F,G	161.0 ± 27.4<br>A,B,C,D,E	160.5 ± 27.7<br>A,B,C,D,E
HDL-c	M0	59.5 ± 13.3<br>C,D	62.6 ± 13.4<br>C,D,E	66.2 ± 13.3<br>A,B,E,F,G	66.9 ± 13.3<br>A,B,E,F,G	59.7 ± 13.5<br>B,C,D	59.7 ± 13.4<br>B,C,D	60.5 ± 13.4<br>C,D
	M1	59.5 ± 13.3<br>B,C,D	62.8 ± 13.4<br>A,D,E,F	66.1 ± 13.3<br>A,E,F,G	66.9 ± 13.3<br>A,B,E,F,G	59.6 ± 13.2<br>B,C,D	59.8 ± 13.2<br>B,C,D	60.6 ± 13.4<br>C,D
	M2	58.6 ± 12.6<br>B,C,D	62.8 ± 12.7<br>A,D,E	65.7 ± 12.5<br>A,E,F,G	66.9 ± 12.6<br>A,B,E,F,G	60.2 ± 12.5<br>B,C,D	59.4 ± 12.6<br>B,C,D	60.4 ± 12.6<br>C,D
LDL-c	M0	113.6 ± 24.0<br>B,C,D,E,F,G	101.9 ± 24.0<br>A,D,F,G	101.2 ± 24.0<br>A,D,F,G	90.4 ± 24.0<br>A,B,C,E	97.2 ± 24.0<br>A,D,G	93.7 ± 24.0<br>A,B,C	90.1 ± 24.0<br>A,B,C,E
	M1	113.6 ± 24.0<br>B,C,D,E,F,G	102.0 ± 24.3<br>A,D,F,G	101.1 ± 24.1<br>A,D,F,G	90.4 ± 24.3<br>A,B,C,E	97.2 ± 24.0<br>A,D,G	93.7 ± 24.0<br>A,B,C	90.2 ± 24.3<br>A,B,C,E
	M2	114.3 ± 23.9<br>B,C,D,E,F,G	102.1 ± 24.2<br>A,D,E,F,G	101.4 ± 23.8<br>A,D,F,G	90.4 ± 23.9<br>A,B,C,E	96.8 ± 23.9<br>A,B,D,G	93.9 ± 23.8<br>A,B,C	90.4 ± 24.1<br>A,B,C,E
TG	M0	57.5 ± 31.0<br>E,F	57.2 ± 30.9<br>E,F	54.8 ± 31.0<br>E,F	60.7 ± 30.9<br>E,F	68.7 ± 31.0<br>A,B,C,D,G	71.0 ± 30.9<br>A,B,C,D,G	62.0 ± 30.8<br>,E,F
	M1	57.8 ± 30.7<br>E,F	55.8 ± 31.1<br>E,F	55.4 ± 30.7<br>E,F	61.1 ± 30.6<br>E,F	69.0 ± 30.7<br>A,B,C,D,G	70.8 ± 30.6<br>A,B,C,D,G	60.9 ± 31.0<br>E,F
	M2	60.3 ± 28.2<br>E,F	55.9 ± 28.<br>E,F	56.4 ± 28.2<br>E,F	61.0 ± 28.2<br>E,F	67.6 ± 28.3<br>A,B,C,D,G	71.7 ± 28.2<br>A,B,C,D,G	61.6 ± 28.4<br>E,F
Non-HDL-c	M0	125.3 ± 26.2<br>B,C,D,E,F,G	113.3 ± 26.1<br>A,D,F,G	112.2 ± 26.1<br>A,D,F,G	102.6 ± 26.2<br>A,B,C,E	109.5 ± 26.0<br>A,D,F,G	101.2 ± 26.0<br>A,B,C,E	99.8 ± 26.1<br>A,B,C,E
	M1	125.3 ± 26.2<br>B,C,D,E,F,G	113.2 ± 26.4<br>A,D,F,G	112.2 ± 26.1<br>A,D,F,G	102.6 ± 26.2<br>A,B,C,E	109.5 ± 26.0<br>A,D,F,G	101.2 ± 26.0<br>A,B,C,E	99.9 ± 26.5<br>A,B,C,E
	M2	126.5 ± 25.5<br>B,C,D,E,F,G	113.3 ± 25.7<br>A,D,F,G	112.6 ± 25.4<br>A,D,F,G	102.5 ± 25.6<br>A,B,C,E	108.9 ± 25.6<br>A,D,F,G	101.6 ± 25.4<br>A,B,C,E	100.1 ± 25.7<br>A,B,C,E
REM-c	M0	14.2 ± 4.1<br>E,F	14.0 ± 4.2<br>E,F	13.6 ± 4.3<br>E,F	14.2 ± 4.3<br>E,F	15.4 ± 4.0<br>A,B,C,D,G	15.6 ± 4.2<br>A.B.C.D.G	14.4 ± 4.2<br>E.F
	M1	14.2 ± 4.1<br>E,F	13.8 ± 4.2<br>E,F	13.7 ± 4.1<br>E,F	14.3 ± 4.3<br>E,F	15.5 ± 4.0<br>A,B,C,D,G	15.6 ± 4.2<br>A,B,C,D,G	14.2 ± 4.2<br>E,F
	M2	14.6 ± 3.8<br>F	13.8 ± 3.8<br>E,F	13.8 ± 3.8<br>E,F	14.3 ± 4.0<br>E,F	15.3 ± 3.7<br>B,C,D,G	15.7 ± 3.7<br>A,B,C,D,G	14.3 ± 3.8<br>E,F

MO: Crude model; M1: Adjusted by age and sex; M2: Adjusted by age, sex and BMI.
Data is presented as mean (mg/dl) ± standard deviation.
All ANCOVA models displayed were statistically significant (p<.001), uppercase characters letters indicate statistical significance (p < 0.05) between survey year for post-hoc tests using the Bonferroni.
Abbreviations: HDL-c: high density lipoprotein cholesterol; LDL-c: low density lipoprotein cholesterol; TC: total cholesterol; TG: triglycerides; REM-c: remnant cholesterol.

eTable 4. Levels of Glycemic Parameters From 2004 to 2022

© 2025 Sequí-Domínguez I et al. *JAMA Network Open*.

		2004 (A)	2010 (B)	2018 (C)	2022 (D)
	n	1119	1157	559	557
Fasting plasma glucose (mg/dl)	M0	86.2 ± 7.0 B,C,D	83.6 ± 7.1 A,C,D	87.6 ± 7.1 A,B,D	84.8 ± 7.0 A,B,C
	M1	86.3 ± 7.0 B,C,D	83.7 ± 6.7 A,C,D	87.6 ± 6.8 A,B,D	84.3 ± 7.0 A,B,C
	M2	86.3 ± 7.0 B,C,D	83.6 ± 6.7 A,C,D	87.6 ± 6.8 A,B,D	84.3 ± 7.0 A,B,C
Insulin (mcIU/ml)	M0	6.3 ± 5.3 B,C,D	8.0 ± 5.4 A	8.2 ± 5.3 A	8.7 ± 5.2 A
	M1	6.3 ± 5.3 B,C,D	8.0 ± 5.1 A	8.2 ± 5.1 A	8.5 ± 5.2 A
	M2	6.4 ± 4.6 B,C,D	7.8 ± 4.4 A	8.4 ± 4.4 A	8.7 ± 4.6 A

MO: Crude model; M1: Adjusted by age and sex; M2: Adjusted by age, sex and BMI.
Data is presented as mean ± standard deviation.
All ANCOVA models displayed were statistically significant (p<.001), uppercase characters letters indicate statistical significance (p < 0.05) between survey year for post-hoc tests using the Bonferroni.

eTable 5. Levels in Blood Pressure Parameters From 1992 to 2022

		1992 (A)	1996 (B)	1998 (C)	2004 (D)	2010 (E)	2018 (F)	2022 (G)
	n	305	307	276	1119	1157	559	554
SBP	M0	113.5 ± 9.6 B,C,D,E,F,G	119.7 ± 9.7 A,C,D,E,F,G	109.5 ± 9.6 A,B,D,E,F,G	107.0 ± 9.7 A,B,C,E,F,G	101.2 ± 9.5 A,B,C,D,F	98.8 ± 9.7 A,B,C,D,E,G	101.0 ± 9.7 A,B,C,D,F
	M1	113.7 ± 9.6 B,C,D,E,F,G	118.9 ± 9.7 A,C,D,E,F,G	109.8 ± 9.5 A,B,D,E,F,G	107.2 ± 9.7 A,B,C,E,F,G	101.3 ± 9.5 A.B,C,D,F	98.8 ± 9.5 A,B,C,D,E,G	100.3 ± 9.7 A,B,C,D
	M2	114.1 ± 9.3 B,C,D,E,F,G	118.5 ± 9.3 A,C,D,E,F,G	110.0 ± 9.3 A,B,D,E,F,G	107.2 ± 9.4 A,B,C,E,F,G	101.0 ± 9.2 A,B,C,D,F	99.0 ± 9.2 A,B,C,D,E,G	100.4 ± 9.4 A,B,C,D
DBP	M0	70.4 ± 7.2 B,D,E,F,G	64.3 ± 7.2 A,C,G	68.7 ± 7.1 B,D,E,F,G	65.1 ± 7.3 A,C,E,F,G	62.4 ± 7.1 A,C,D,F,G	63.6 ± 7.3 A,C,D,E,G	60.7 ± 7.3 A,B,C,D,E,F
	M1	70.5 ± 7.2 B,D,E,F,G	64.0 ± 7.3 A,C,G	68.8 ± 7.1 B,D,E,F,G	65.1 ± 7.3 A,C,E,F,G	62.4 ± 7.1 A,C,D,F,G	63.6 ± 7.1 A,C,D,E,G	60.5 ± 7.3 A,B,C,D,E,F
	M2	70.8 ± 7.0 B,C,D,E,F,G	63.9 ± 7.0 A,C,G	69.0 ± 7.0 B,D,E,F,G	65.1 ± 7.0 A,C,E,F,G	62.2 ± 6.8 A,C,D,F,G	63.8 ± 6.9 A,C,D,E,G	60.6 ± 7.1 A,B,C,D,E,F

M0: Crude values; M1: Adjusted by age, sex; M2: Adjusted by age, sex and BMI
Data is presented as mean (mmHg) ± standard deviation.
All ANCOVA models displayed were statistically significant (p<.001), uppercase characters letters indicate statistical significance (p < 0.05) between survey year for post-hoc tests using the Bonferroni.
Abbreviations: DBP: diastolic blood pressure; SBP: systolic blood pressure.

© 2025 Sequí-Domínguez I et al. *JAMA Network Open*.

eTable 6. Levels of Lipid, Glycemic, and Blood Pressure Parameters From 1992 to 2022[a]

**Measurement**	**1992 (n = 305)**	**1996 (n = 307)**	**1998 (n = 276)**	**2004 (n = 1119)**	**2010 (n = 1157)**	**2018 (n = 559)**	**2022 (n = 557)**
Lipid profile							
TC, mg/dL	184.8 (27.4)[b]	176.1 (27.7)[c]	178.4 (27.4)[d]	169.4 (27.4)[e]	169.0 (27.4)[e]	161.0 (27.4)[f]	160.5 (27.7)[f]
HDL-C, mg/dL	58.6 (12.6)[g]	62.8 (12.7)[h]	65.7 (12.5)[i]	66.9 (12.6)[j]	60.2 (12.5)[g]	59.4 (12.6)[g]	60.4 (12.6)[k]
LDL-C, mg/dL	114.3 (23.9)[l]	102.1 (24.2)[c]	101.4 (23.8)[m]	90.4 (23.9)[n]	96.8 (23.9)[o]	93.9 (23.8)[p]	90.4 (24.1)[n]
TG, mg/dL	60.3 (28.2)[q]	55.9 (28.0)[q]	56.4 (28.2)[q]	61.0 (28.2)[q]	67.6 (28.3)[r]	71.7 (28.2)[r]	61.6 (28.4)[q]
Non-HDL-C, mg/dL	126.2 (25.1)[l]	113.3 (24.5)[m]	112.7 (24.2)[m]	102.5 (24.1)[n]	108.8 (24.3)[m]	101.6 (24.0)[n]	100.1 (24.3)[n]
REM-C, mg/dL	14.6 (3.8)[s]	13.8 (3.8)[q]	13.8 (3.8)[q]	14.3 (4.0)[q]	15.3 (3.7)[t]	15.7 (3.7)[r]	14.3 (3.8)[q]
Glycemic parameters							
FPG, mg/dL	NA	NA	NA	86.3 (7.0)[u]	83.6 (6.7)[u]	87.6 (6.8)[u]	84.3 (7.0)[u]
Insulin, µIU/mL	NA	NA	NA	6.4 (4.6)[u]	7.8 (4.4)[v]	8.4 (4.4)[v]	8.7 (4.6)[v]
Blood pressure							
SBP, mm Hg	114.1 (9.3)[u]	118.5 (9.3)[u]	110.0 (9.3)[u]	107.2 (9.4)[u]	101.0 (9.2)[w]	99.0 (9.2)[u]	100.4 (9.4)[w]
DBP, mm Hg	70.8 (7.0)[u]	63.9 (7.0)[x]	69.0 (7.0)[b]	65.1 (7.0)[y]	62.2 (6.8)[z]	63.8 (6.9)[aa]	60.6 (7.1)[u]

Abbreviations, DBP, diastolic blood pressure; FPG, fasting plasma glucose; HDL-C, high density lipoprotein cholesterol; LDL-C, low density lipoprotein cholesterol; NA, not applicable; REM-C, remnant cholesterol; SBP, systolic blood pressure; TC, total cholesterol; TG, triglycerides.
SI conversions: To convert FPG to millimoles per liter, multiply by 0.0555; HDL-C to millimoles per liter, multiply by 0.259; insulin to picomoles per liter, multiply by 6.945; LDL-C to millimoles per liter, multiply by 0.259; TC to millimoles per liter, multiply by 0.259; TG to millimoles per liter, multiply by 0.113.
[a]Values adjusted by age, sex and BMI. All analysis of covariance models displayed were statistically significant ($P < .001$), superscript letters indicate statistical significance ($P < .05$) between survey year for post hoc tests using the Bonferroni comparisons.
[b]Significantly different from 1996, 2004, 2010, 2018, 2022.
[c]Significantly different from 1992, 2004, 2010, 2018, 2022.
[d]Significantly different from 2004, 2010, 2018, 2022.
[e]Significantly different from 1992, 1996, 1998, 2018, 2022.
[f]Significantly different from 1992, 1996, 1998, 2004, 2010.
[g]Significantly different from 1996, 1998, 2004.
[h]Significantly different from 1992, 2004, 2010.
[i]Significantly different from 1992, 2010, 2018, 2022.
[j]Significantly different from 1992,1996, 2010, 2018, 2022.
[k]Significantly different from 2004,2010.
[l]Significantly different from 1996, 1998,2004, 2010, 2018, 2022.
[m]Significantly different from 1992, 2004, 2018, 2022.
[n]Significantly different from 1992, 1996, 1998, 2010.
[o]Significantly different from 1992, 1996, 2004, 2022.
[p]Significantly different from 1992, 1996, 1998.
[q]Significantly different from 2010, 2018.
[r]Significantly different from 1992,1996, 1998, 2004, 2022.
[s]Significantly different from 2018.

© 2025 Sequí-Domínguez I et al. *JAMA Network Open*.

[t]Significantly different from 1996, 1998, 2004, 2022.
[u]Significantly different from all other survey years.
[v]Significantly different from 2004.
[w]Significantly different from 1992,1996, 1998, 2004, 2018.
[x]Significantly different from 1992, 1998, 2022.
[y]Significantly different from 1992, 1998, 2010, 2018, 2022.
[z]Significantly different from 1992, 1998, 2004, 2018, 2022.
[aa]Significantly different from 1992, 1998, 2004, 2010, 2022.

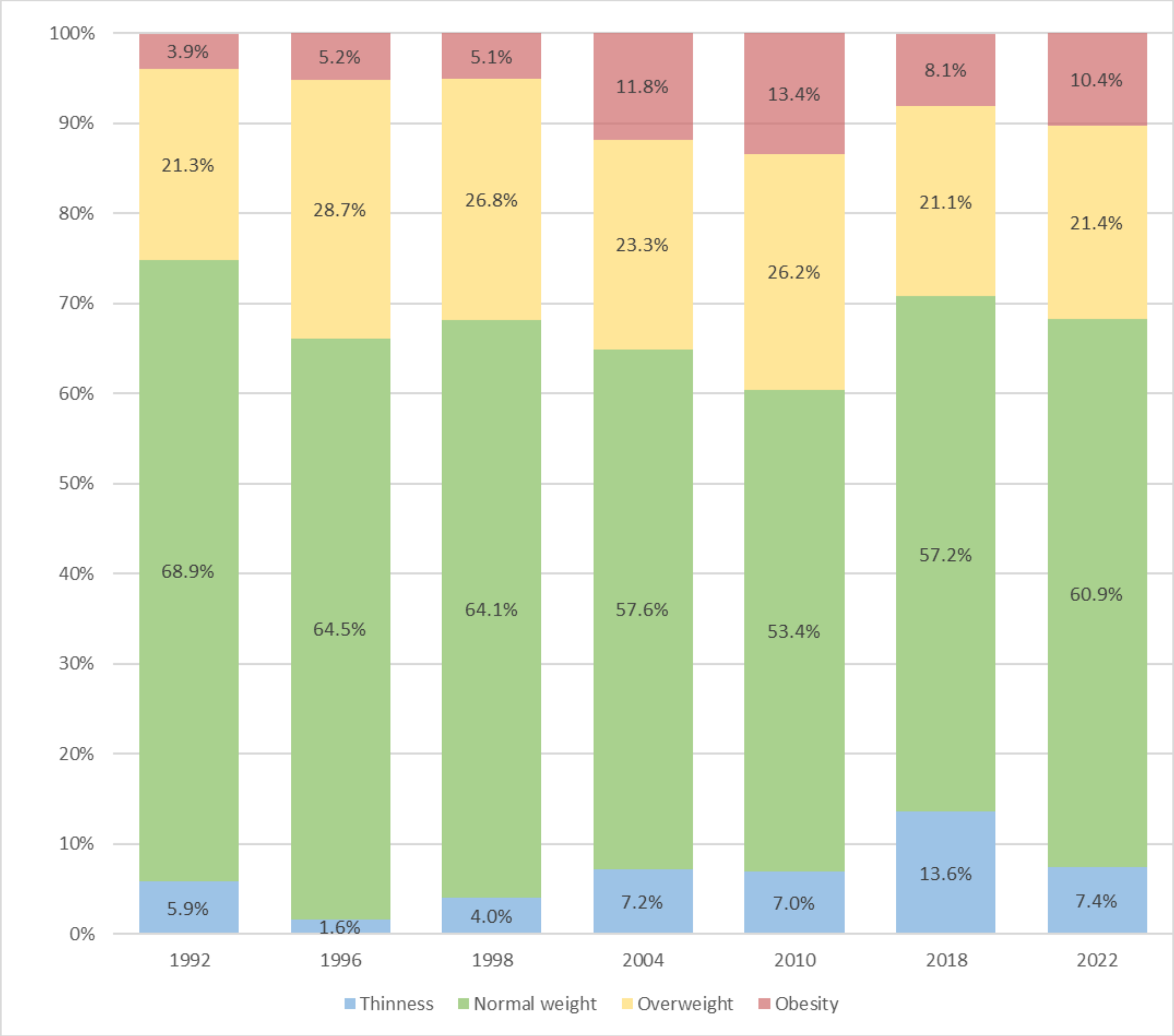

eFigure 1. Prevalence and Patterns of Underweight, Normal Weight, Overweight, and Obesity From 1992 to 2022

© 2025 Sequí-Domínguez I et al. *JAMA Network Open*.

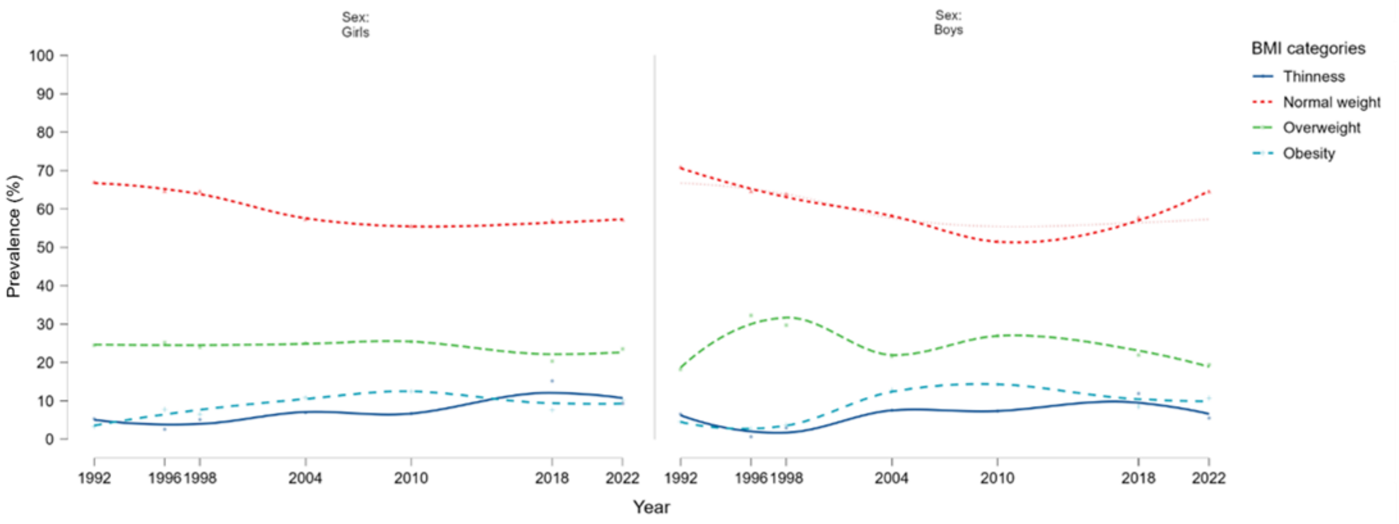

eFigure 2. Secular Patterns in Prevalence of Underweight, Normal Weight, Overweight, and Obesity From 1992 to 2022 by Sex

Abbreviations: BMI: body mass index

© 2025 Sequí-Domínguez I et al. *JAMA Network Open*.

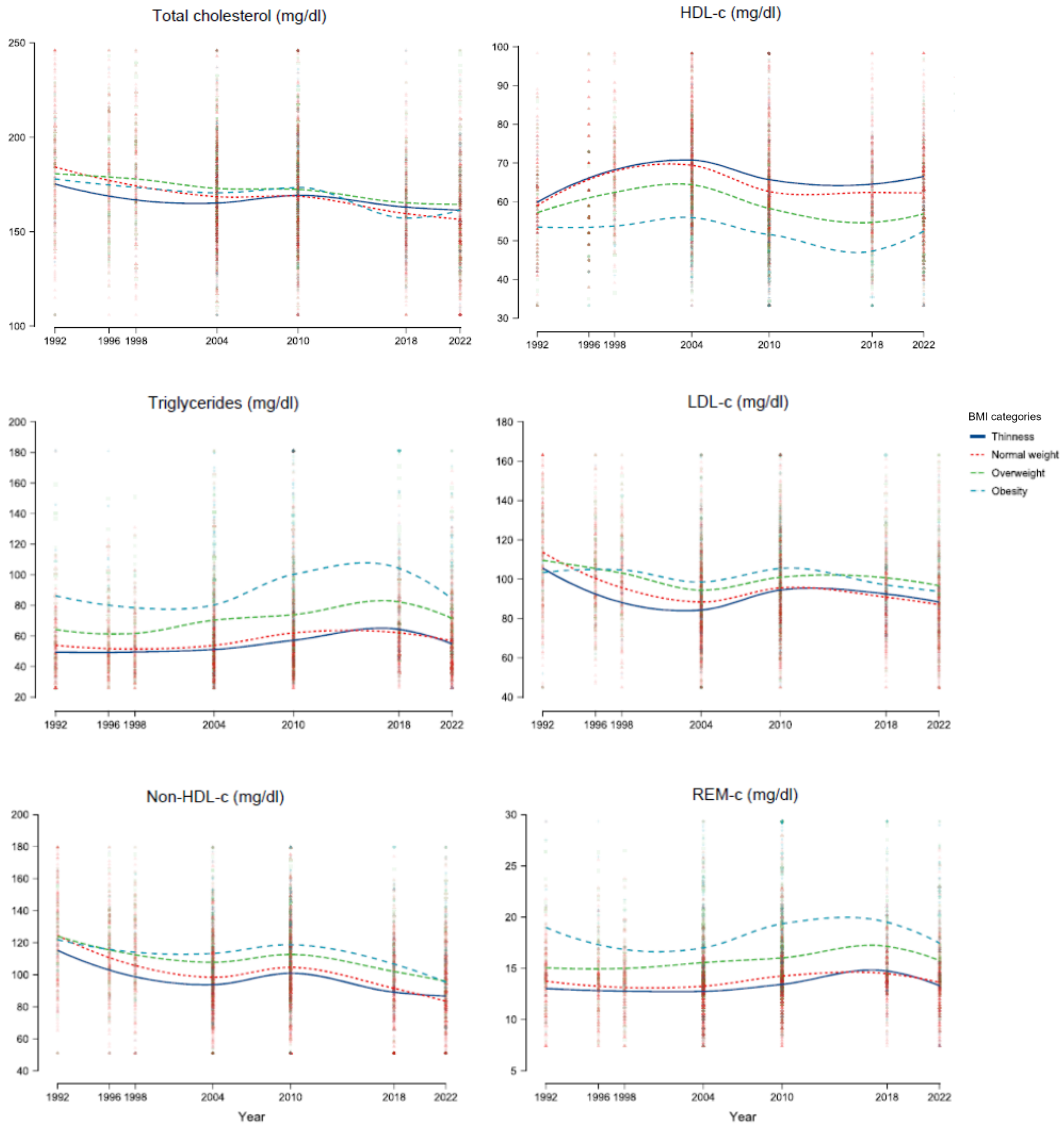

eFigure 3. Locally Weighted Regression (LOESS) Patterns of Lipid Parameters From 1992 to 2022 by IOTF Categories

Intervals represent standard deviations.
All displayed patterns were statistically significant (p<.001)
Abbreviations: BMI: body mass index; HDL-c: high density lipoprotein cholesterol; LDL-c: low density lipoprotein cholesterol; REM-c: remnant cholesterol

© 2025 Sequí-Domínguez I et al. *JAMA Network Open*.

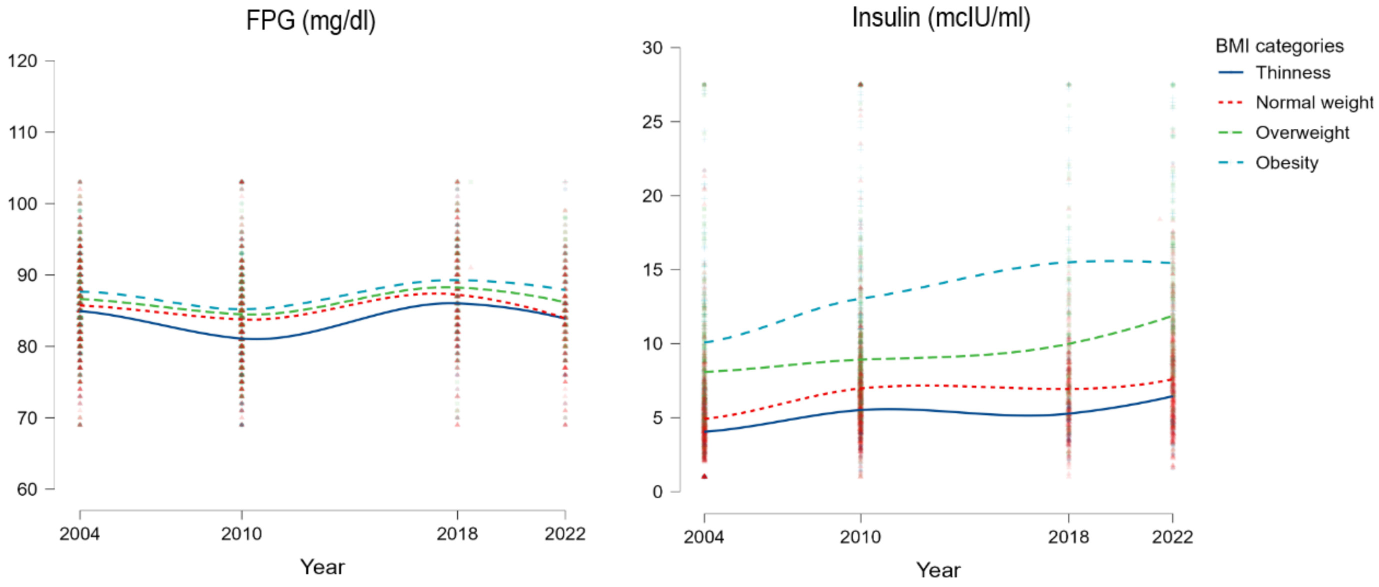

eFigure 4. Locally Weighted Regression (LOESS) Pattern of Fasting Plasma Glucose (FPG) and Insulin Levels From 2004 to 2022

All displayed patterns were statistically significant (p < .001)

Intervals represent standard deviations.

Abbreviations: BMI: Body mass index; FPG: fasting plasma glucose

© 2025 Sequí-Domínguez I et al. *JAMA Network Open*.

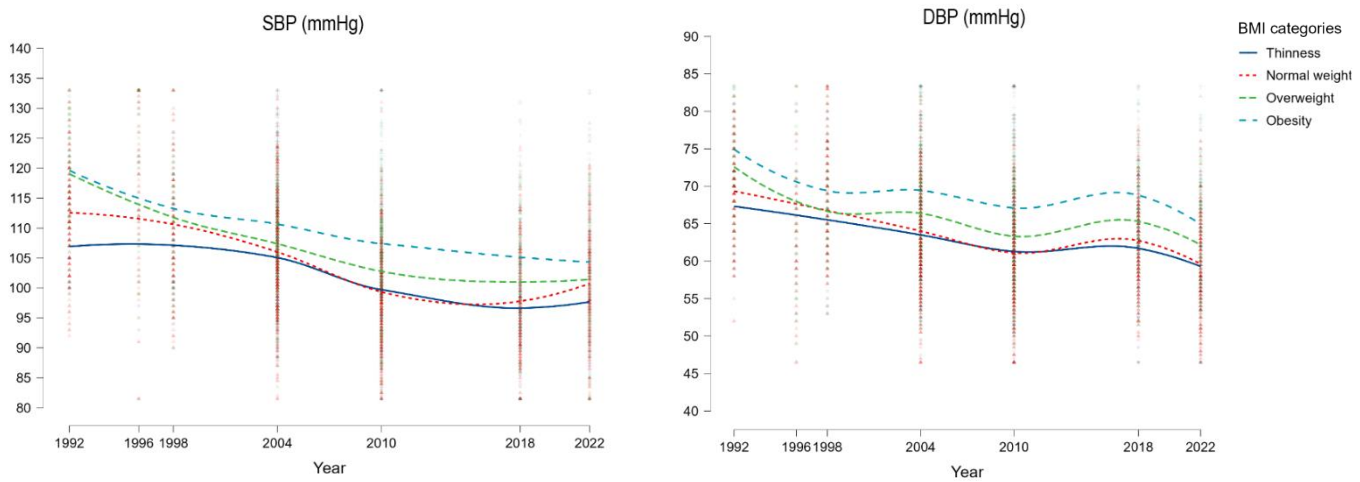

eFigure 5. Locally Weighted Regression (LOESS) Patterns of systolic Blood Pressure (SBP) and Diastolic Blood Pressure (DBP) From 1992 to 2022

All displayed patterns were statistically significant (p < .001)

Intervals represent standard deviations.

Abbreviations: DBP: diastolic blood pressure; SBP: systolic blood pressure

© 2025 Sequí-Domínguez I et al. *JAMA Network Open*.
