# Supplementary material for: Body Composition and Cardiometabolic Risk in Children
Source: JAMA Netw Open. 2025 Oct 2;8(10):e2535004. doi: 10.1001/jamanetworkopen.2025.35004 (PMC12492057; doi:10.1001/jamanetworkopen.2025.35004)
Supplement: Supplement 2. — Data Sharing Statement [file jamanetwopen-e2535004-s002.pdf]

Data Sharing Statement

Sequí-Domínguez. Body Composition and Cardiometabolic Risk in Children in Spain, 1992–2022. *JAMA Netw Open*. Published October 02, 2025.
doi:10.1001/jamanetworkopen.2025.35004

Data
Data available: Yes
Data types: Deidentified participant data
How to access data: The data that support the findings of this study are available on request from the corresponding author. The data are not publicly available due to privacy or ethical restrictions.
When available: With publication

Supporting Documents
Document types: None

Additional Information
Who can access the data: Researchers whose proposed use of the data has been approved
Types of analyses: Any purpose approved
Mechanisms of data availability: With investigator support after approval of a proposal.
